# Effects of dignity therapy on psychological distress and wellbeing of palliative care patients and family caregivers – a randomized controlled study

**DOI:** 10.1186/s12904-024-01408-4

**Published:** 2024-03-14

**Authors:** Annina Seiler, Manuel Amann, Caroline Hertler, Sebastian M. Christ, Markus Schettle, Barbara Meier Kaeppeli, Judith Jung-Amstutz, Christel Nigg, Bernhard C. Pestalozzi, Patrick Imesch, Reinhard Dummer, David Blum, Josef Jenewein

**Affiliations:** 1 Department of Radiation Oncology, Competence Center for Palliative Care, University Hospital Zurich and University of Zurich, Rämistrasse 100, 8091 Zurich, Switzerland; 2 Department of Gynecology, University Hospital Zurich, Zurich, Switzerland; 3 Klinik Susenberg, Zurich, Switzerland; 4 Department of Medical Oncology and Hematology, University Hospital Zurich and University of Zurich, Zurich, Switzerland; 5 Department of Dermatology, University Hospital Zurich and University of Zurich, Zurich, Switzerland; 6 Privatklinik Hohenegg, Meilen, Switzerland; 7 University of Zurich, Zurich, Switzerland

**Keywords:** Dignity therapy, Psychological distress, Patients, Family caregivers, Palliative care

## Abstract

**Background:**

This study extended the original Dignity Therapy (DT) intervention by including partners and family caregivers (FCs) of terminally-ill cancer patients with the overall aim of evaluating whether DT can mitigate distress in both patients nearing the end of life and their FCs.

**Methods:**

In this multicenter, randomized controlled trial (RCT), a total of 68 patients with life expectancy < 6 months and clinically-relevant stress levels (Hospital Anxiety Depression total score; HADS_tot_ ≥ 8) including their FCs were randomly assigned to DT, DT + (including their FCs), or standard palliative care (SPC) in a 1:1:1 ratio. Study participants were asked to complete a set of questionnaires pre- and post-intervention.

**Results:**

The coalesced group (DT and DT +) revealed a significant increase in patients’ perceived quality of life (FACIT-Pal-14) following the intervention (mean difference 6.15, SD = 1.86, *p* < 0.01). We found a statistically significant group-by-time interaction effect: while the HADS_tot_ of patients in the intervention group remained stable over the pre-post period, the control group’s HADS_tot_ increased (F = 4.33, df = 1, 82.9; *p* < 0.05), indicating a protective effect of DT. Most patients and their FCs found DT useful and would recommend it to other individuals in their situation.

**Conclusions:**

The DT intervention has been well-received and shows the potential to increase HRQoL and prevent further mental health deterioration, illness burden and suffering in terminally-ill patients. The DT intervention holds the potential to serve as a valuable tool for facilitating end-of-life conversations among terminally-ill patients and their FCs. However, the implementation of DT within the framework of a RCT in a palliative care setting poses significant challenges. We suggest a slightly modified and less resource-intensive version of DT that is to provide the DT inventory to FCs of terminally-ill patients, empowering them to ask the questions that matter most to them over their loved one’s final days.

**Trial registration:**

This study was registered with Clinical Trial Registry (ClinicalTrials.gov -Protocol Record NCT02646527; date of registration: 04/01/2016). The CONSORT 2010 guidelines were used for properly reporting how the randomized trial was conducted.

**Supplementary Information:**

The online version contains supplementary material available at 10.1186/s12904-024-01408-4.

## Background

Terminal illnesses bring substantial physical and psychological challenges, including multiple physical symptoms and progressive physical deterioration that may contribute to psychological distress, anxieties, and depressive symptoms [42]. Depressive symptoms, death anxiety, and perceived loss of control, dignity, meaning, and purpose are important risk factors for the wish to hasten death [14, 32, 33, 43]. The most commonly reported reasons for wanting hastened death and medical aid in dying (MAID) are not physical symptoms, but existential and dignity-related concerns like illness-related suffering, lost autonomy and control, lost meaning in life, or the feeling of being a burden [20, 53, 56]. Similar results have been found for studies conducted in Switzerland. The majority of individuals who sought assisted suicide in Switzerland did so due to existential distress, fear of loss of control [19], and loss of dignity and autonomy [52]. Research into the determinants associated with the wish for hastened death highlight the critical importance of personalized care plans and interventions that prioritize promoting dignity in individuals with advanced cancer, as a way to mitigate such patients’ inclination towards hastened death [35].

Dignity, originally a philosophical and/or spiritual construct, is widely accepted as a fundamental cornerstone of palliative care. Within the context of healthcare, dignity has been defined as a state in which a patient is able to live in alignment with their individual values and standards [4]. Specifically, dying with dignity has been characterized by a set of essential components, which include recognition of one’s inherent human values and worth, being cared for with empathy and respect, having a voice regarding one’s process of dying, experiencing minimal physical and emotional suffering, safeguarding one’s privacy, being emotionally connected with others, bringing personal affairs to resolution, and having access to spiritual support [40]. Some aspects of dignity – such as symptom management, privacy, respect, and a calm environment – can be provided effectively by appropriate and established means of standard palliative care [27]. However, other aspects of dignity – like not feeling dehumanized and retaining a sense of self, experiencing meaningful relationships, and achieving existential fulfillment – are more challenging to address and necessitate individual-level interventions that are specifically tailored to each patient’s unique needs.

In recent years, a variety of dignity-conserving interventions have been proposed to address existential concerns at the end of life. One prominent approach is ‘Dignity Therapy’, a brief, individualized psychotherapeutic intervention designed to improve quality of life and mitigate the existential suffering of terminally-ill patients by addressing dignity-promoting factors [12]. Dignity Therapy involves creating a legacy document (‘generativity document’) that captures each patient’s life story, values and hopes for the future. At present, DT has been evaluated in the context of specialized palliative care settings across different countries and proven to be highly feasible, to increase patients’ quality of life and sense of dignity, and to be helpful to their families [14, 16, 18, 22, 34]. However, only a few studies have identified a significant reduction in psychological distress or depression following DT [15, 26], while most of the studies failed to demonstrate such effect [6, 14, 21]. Of note, the levels of anxiety and distress at baseline varied considerably across studies. For example, Chochinov et al. [14] reported significantly lower anxiety and depression levels at baseline than Julião et al. [26] (HADS anxiety: 5.2 versus 10.0,HADS depression 5.9 vs 14.0). The variability in HADS baseline levels across different studies may be attributed to methodological and measurement issues, including floor effects in assessing psychological distress, anxiety, and depression. These issues can limit the interpretation of study results regarding the efficacy of the DT. This methodological concern underscores the importance of employing a controlled trial design in future studies.

Furthermore, there is only a limited body of literature that has examined the impact of DT on patients’ caregivers and families. Palliative care patients often have partners or family caregivers (FCs) who accompany them throughout their disease journey. Due to the high degree of interdependence that exists between patients and their partners, partners may suffer equally from high-level distress, impaired quality of life, and poor health outcomes [17, 37]. Despite the pivotal role that caregivers play in palliative care, FCs of terminally-ill individuals are rarely included in dignity-promoting interventions [57]. Therefore, further research is warranted to better understand the potential effects of DT on the FCs of palliative care patients.

Taking into account these prior limitations, this study’s innovation is that it extends the original DT intervention by including the FCs of terminally-ill cancer patients at a tertiary care center and in hospice care. The specific aims were: (1) to assess the feasibility and acceptability of dignity therapy (DT) interventions that included FCs of terminally-ill cancer patients receiving acute hospital care or hospice referrals; (2) to examine whether DT can mitigate distress and depression in individuals nearing their end of life; and (3) to determine whether DT can reduce bereavement-related distress in their FCs.

## Methods

This study, conducted across multiple centers, was a randomized controlled trial conducted over a 7-year period (recruitment period: 2015 – 2021) designed to investigate the effectiveness of a DT intervention in patients and their FCs (partners or close family members) in three groups – patients receiving standard palliative care and DT (the DT group), patients receiving standard palliative care and DT along with their FCs (DT +) – relative to a control group consisting of patients who received standard palliative care without DT (SPC). Patients’ FCs were included into the study and asked to fill in a set of questionnaires. Written informed consent was obtained from each participant (patients and their FCs) prior to study participation. Participants were made aware of their freedom to withdraw from the study at any point in time with no unfavorable consequences to them. This study was approved by the Ethics Committee of the Canton Zurich (KEK), Switzerland (KEK-ZH-Nr. PB_2016-01275). The study was also registered with ClinicalTrials.gov (Protocol Record NCT02646527). Furthermore, all procedures adhered to the World Health Organization's Declaration of Helsinki. The study implemented the CONSORT (Consolidated Standards of Reporting Trials) statement as a framework for reporting and conducting the research (the CONSORT 2010 checklist can be found in an Additional file 1) [47].

### Participants

Study participants were recruited from three study sites, which included the University Hospital Zurich with various medical institutions: the Competence Center for Palliative Care (JJ/DB), the Department of Medical Oncology and Hematology (BP), the Department of Gynecology (PI), and the Department of Dermatology (RD). Additionally, study participants were recruited from the Susenberg Clinic (CN) and the Zurich Lighthouse Hospice (JJ). The study procedures and adherence to ethical guidelines at each study site were overseen by a designated study advisor (see author abbreviation in brackets). All three clinics are situated in close proximity. To be eligible for the study, individuals had to be at least 18 years of age, be diagnosed with terminal cancer (expected life-expectancy ≤ 6 months), be in a relationship with a partner for at least two years or have an informal caregiver, and have a Hospital Anxiety and Depression Scale (HADS) score of at least eight points during pre-screening. Participants with cognitive impairments such as dementia or delirium, those who were too ill to fulfill the study requirements, and those who were unable to read and speak German were excluded from the study.

### Implementation of dignity therapy

The Dignity Therapy was implemented across the three study sites using a systematic and structured approach. The implementation process encompassed several key steps, including training and education of various health professionals, including physicians, nurses, chaplains, social workers, clinical psychologists, physiotherapists, and occupational therapists at each study site. The training involved a one-hour teaching lecture on the Dignity Therapy intervention, followed by a presentation of the study and its procedures. During these training sessions, essential resources, such as study flyers and the Dignity Therapy inventory were provided to each study site.

### Procedures

While being mindful of the patient's vulnerability, in an initial encounter with the patient and their FC, DT was introduced briefly as a potential supportive care option by a physician or a nurse working on one of the three study sites. Potential patients and their FCs were provided with complete information regarding the study by the study coordinator. Because it might be difficult to talk about the end of life or the possibility of death, eligible study participants were given enough time (at least 24 h) to consider their study participation and to think about the content of the DT interview. If the patients and their FCs showed interest in DT, the research coordinator followed up with them to schedule a research visit. Once the patient and their FC provided their written consent to participate, a pre-assessment was performed to screen for each patient’s anxiety and depression levels. Only those patients with clinically-significant stress levels, defined as a total HADS_tot_ ≥ 8 were considered eligible for the study. Study participants were randomly assigned to either DT + , DT, or SPC in a 1:1:1 ratio using the extended stratified block without list algorithm offered by secuTrial [49].

**Fig. 1:**
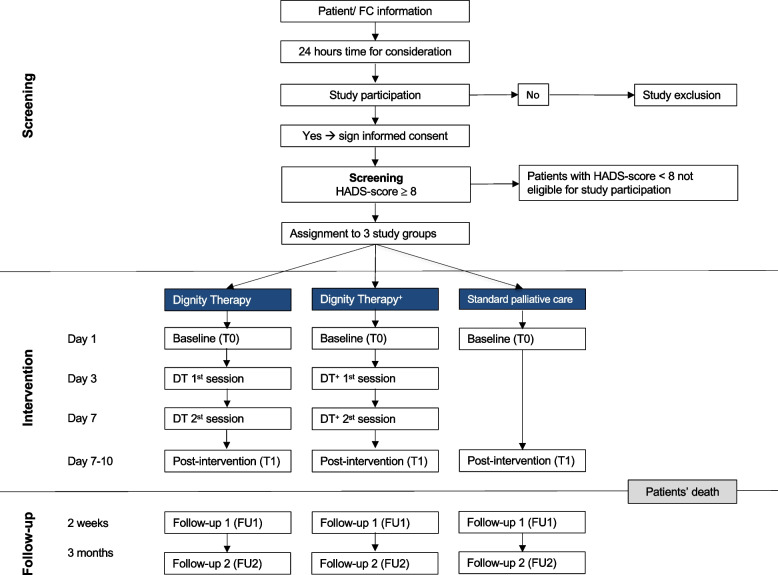
Study flow chart

Prior to and after the DT intervention, participants and their FCs were required to complete a standardized set of questionnaires. Primary and secondary outcomes were evaluated in patients and FCs at baseline (T0) and one-week post intervention (T1). In addition, FCs were recontacted two weeks (T2) and three months (T3) following their partner’s death (Fig. 1).

### Dignity therapy

Dignity Therapy is a brief, individual, dignity-promoting intervention that enables persons to perform a guided appraisal of meaningful moments and memories in their life while creating a unique written legacy for their families (i.e., ‘generativity document’) [10]. In our study, the DT intervention included three sessions: a brief introduction, the main session, and a final session. During the main session (approximately 60 min in duration), patients, either alone or accompanied by their partner or caregivers, were engaged in a dignity-enhancing conversation guided by a trained therapist, employing a semi-structured interview protocol to elicit the desired thoughts and expressions that patients wished to communicate to their loved ones. In the DT + group, the patients and their family caregivers were instructed that the patient's narrative holds primary importance. However, if the patient desired, the family caregiver could contribute to the patient’s narrative for entirety of the patient’s story. The semi-structured interview guideline is adaptable to patients’ individual needs and specifications and may involve reflections on personal history, important achievements, roles, hopes, wishes, dreams, and anything else the study participants wished to be remembered or shared about themselves. The patients’ narrations were recorded on audiotape, transcribed verbatim, and edited to produce a readable and clear narrative: *the generativity document*. During the final session (approximately 60 min in duration), the generativity document was read aloud to the patient to ensure accuracy and make any necessary corrections. The final version of the document was then given to each patient to share with their significant other or family.

### Training and education of DT intervention therapists

One of the authors, AS, completed a DT training workshop with the originator, Harvey Max Chochinov (University Hospital Basel, Switzerland, 2018) and with Sandra Mai and Jan Gramm (Mainz, Frankfurt, 2015), respectively. AS trained a total of four psychologists with more than 3 years of clinical experience in the field of psycho-oncology during a one-day workshop including role plays, training videos, and lecture material. In addition, AS provided continued supervision during the recruitment process. DT therapists used a standardized protocol that clearly outlined the steps, procedures, and techniques of the DT intervention to ensure fidelity and consistency of intervention delivery across different sessions and DT therapists.

### Outcomes on acceptability and feasibility

*Acceptability* of DT was assessed using a series of items with 5-point Likert response scales, which allowed participants to rate their DT experience. These items were specifically crafted to evaluate participants' perceptions regarding how helpful DT was for themselves and their partners or caregivers, its capacity to enhance personal meaning, their inclination to recommend DT to other patients and families, and the benefits they obtained from their participation in DT. Likert scales have been utilized extensively in research as robust and valid instruments to measure the attitudes, opinions, and experiences of study participants [54].

The *feasibility* of DT was assessed by measuring the number of visits made by therapists, the time taken by therapists to conduct the interviews, transcribe and edit the generativity documents, and the participants’ dropout rate. The number of visits and time taken by therapists provided insights into the potential burden that DT may impose on healthcare resources. Meanwhile, the drop-out rate was a crucial factor in assessing the feasibility of DT among terminally-ill adults with cancer. This set of feasibility metrics provided valuable information to aid in determining the practicality of integrating DT into routine palliative care.

### Measures

Sociodemographic and clinical data of patients and their partners or caregivers were collected at baseline, including patient age, gender, relationship status, educational level, employment, and religious preferences. Patients’ medical data, including time since their cancer diagnosis, type of cancer, cancer stage, and current therapy regimen, were obtained by reviewing medical records. In addition, patients were asked about their inclination towards hastened death, utilizing a seven-point Likert scale (0 = none, 1 = minimum, 2 = mild, 3 = moderate, 4 = strong, 5 = severe, 6 = extreme).

### Primary outcome

*The Hospital Anxiety and Depression Scale (HADS)* is a validated and widely-used self-report 14-item questionnaire that assesses individuals’ self-perceived levels of depression and anxiety [60]. It contains seven items each for depression and anxiety, each item accompanied by response options ranging from zero to three, with zero being the least and 3 the most indicative of symptoms. It can be used to identify patients with clinically-relevant symptoms of depression and anxiety (probable anxiety: HADS anxiety score > 7; probably depression: HADS depression score > 7). In cancer patients, a HADS total score of ≥ 13 detected 76% with a specificity of 0.60, while a score of ≥ 6 detected 95%, albeit with a lower specificity of 0.21. In clinical studies, the choice between high detection rates or low misclassification rates is crucial when using the HADS [51]. To be eligible for this study, we opted for a middle ground, selecting a cut-off score of a HADS total score ≥ 8.

### Secondary outcomes

*The Distress Thermometer (DTherm)* is a unidimensional tool that utilizes a visual analog scale (VAS) with values ranging from 0 (no distress) to 10 (extreme distress) to assess an individual's self-perceived level of overall distress [44].

*Functional Assessment of Chronic Illness Therapy Palliative Care (FACIT-Pal) and Spiritual Wellbeing Scale (FACIT-SP)* are short, validated instruments used to measure quality of life in palliative care patients. The FACIT-Pal explores symptoms that frequently occur in advanced illness, family and friend relationships, life closure issues, and decision-making and communication abilities [59]. The FACIT-SP [39] specifically assesses spiritual components of quality of life. The present study utilized two subscales from the FACIT-SP: ‘meaning/peace’ and ‘faith’.

*Patient Dignity Inventory—German Version (PDI-G)* is a 25-item questionnaire that has been demonstrated to possess both validity and reliability measuring issues related to dignity at the end of life, including physical symptom distress, loss of sense of worth and meaning, loss of autonomy, anxiety, and uncertainty [13]. Recently, the questionnaire was translated into German [45].

*WHO Quality of Life Questionnaire (WHOQOL-BREF)* is a generic, cross-cultural instrument to measure quality of life. It consists of 26 items covering the domains of physical and psychological health, social relationships, and environment, as well as overall quality of life and general health [2].

*PRISM (Pictorial Representation of Illness and Self Measure)* was developed and validated by Büchi et al. [8] as a simple instrument to visualize illness burden and suffering.

### Statistical methods

The sample size required to achieve an anticipated effect size of a mean difference in HADS total score of 3.0 (with an SD of change 7.7), based on an 80% power and a 5% error, was calculated to be 42 patients in each group. All analyses were performed using the Statistical Package for the Social Sciences (SPSS) version 26.0 software [24]. Descriptive statistics were reported as means (M) and standard deviations (SD), or as counts and percentages (%), as appropriate. All continuous variables were tested for normality using the Shapiro–Wilk’s test. Inter-group comparisons of continuous outcomes were performed using Student's *t*-tests for parametric or Mann–Whitney *U* tests for non-parametric/non-normally distributed data, while categorical variables were compared using Pearson’s-χ^2^ or Fisher’s exact test, as appropriate.

Linear mixed model analysis was utilized to investigate the impact of group (DT, DT + , SPC) and time (baseline T0, post-intervention T1, 2-week FU1, and 3-month follow-up FU2). Our approach to missing data was the assumptions that data was missing at random [5]. Because issues of multiplicity can arise due to repeated measures of the same outcome, a group*time interaction term was also included in the analysis [31]. *Post-hoc* analyses were performed using estimated marginal means to compare time points within groups and between groups at different times. The interaction effect between group and time was evaluated to assess whether improvement varied depending on group membership. The use of linear mixed model analysis with all available outcome data corresponds to intention-to-treat analysis (ITT), which includes all patients who intended to receive treatment at baseline.

In a subsequent step, we merged the DT and DT + groups at each measurement time point to increase statistical power. Doing so, we then utilized the same methodology as before, including linear mixed model analysis for all available outcome data. *Post-hoc* comparisons using estimated marginal means were performed to compare time points within the merged group. All tests were two-tailed and a *p* value < 0.05 was considered statistically significant.

## Results

### Sociodemographic and medical characteristics

The patients’ mean age was 61.5 years (SD = 15.2), while the mean age of their partners was 54.2 years (SD = 16.6) at inclusion. Participants in the SPC (control) group tended to be slightly older than study participants in the DT and DT + groups. Most patients were male (52%), were married (66%), had completed an apprenticeship (19%), and were taken care of by their partner/spouse (78%). Over the entire cohort, the most common underlying malignancy was lung cancer (21%), followed by gynecological cancer (12%), gastrointestinal cancer (12%), and skin cancer (12%). No significant differences were found for baseline characteristics between the different study groups. Due to the small sample size, sociodemographic and disease characteristics are presented as descriptive statistics in Table 1.

**Table 1:** Demographics and medical characteristics of the patients and family caregivers

	**Patients**						**Partners/caregivers**					
	**DT + (*N* = 22)**		**DT (*N* = 26)**		**SPC (*N* = 20)**		**DT + (*N* = 22)**		**DT (*N* = 26)**		**SPC (*N* = 20)**	
	**Mean**	**SD**	**Mean**	**SD**	**Mean**	**SD**	**Mean**	**SD**	**Mean**	**SD**	**Mean**	**SD**
Age	59.4	17.6	59.0	16.0	66.0	12.0	56.8	14.6	51.7	18.6	54.06	16.6
	**%**		**%**		**%**		**%**		**%**		**%**	
Gender N												
Male	59%		46%		40%		55%		54%		65%	
Female	41%		54%		60%		45%		46%		35%	
Marital status												
Married or living with partner	68%		84%		85%		73%		73%		65%	
Divorced or separated	14%		8%		5%		5%		4%		-	
Widowed	-		4%		5%		-		-		-	
Single	18%		4%		5%		15%		4%		25%	
Education												
Obligatory	23%		12%		10%		9%		12%		10%	
Apprenticeship	41%		35%		35%		18%		35%		35%	
High school	9%		4%		5%		-		-		-	
College/university	18%		46%		45%		50%		27%		40%	
Employment status												
Full time	36%		27%		30%		50%		35%		30%	
Part time	23%		19%		30%		14%		19%		25%	
Retired	32%		23%		40%		18%		19%		5%	
Student	5%		-		-		-		4%		5%	
Unemployed due to disability or illness	-		12%		-		5%		-		5%	
Religion												
Catholic	23%		23%		30%		18%		27%		35%	
Reformed	41%		19%		40%		36%		8%		30%	
Other Christian	9%		4%		-		9%		4%		-	
No confession	23%		42%		30%		27%		34%		20%	
Other	4%		12%		-		9%		27%		15%	
Primary disease/cancer site												
Head & neck	5%		8%		10%							
Gynecological	9%		15%		10%							
Lung	23%		19%		20%							
Hematological	5%		8%		5%							
Prostate	-		12%		15%							
Gastrointestinal	5%		19%		10%							
Skin	9%		15%		10%							
Sarcoma	5%		-		5%							
Renal	9%		4%		15%							
Others	30%		-									

*Abbreviations: DT* + Dignity Therapy Patients and Partners, *DT* Dignity Therapy with Patients, *SPC* standard palliative care

### Feasibility

**Fig. 2:**
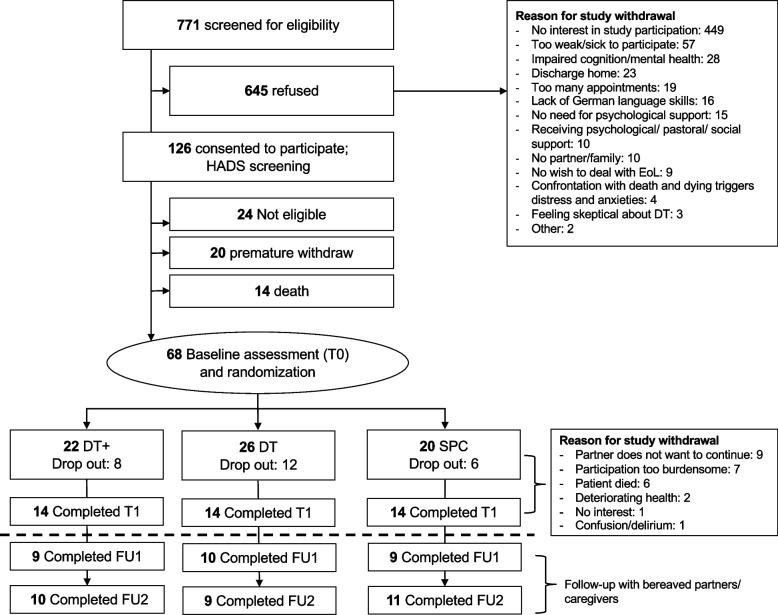
Flow diagram depicting participant recruitment and group allocation

A total of 771 eligible patients were screened for the study between March 2016 and December 2021, among whom 102 individuals were initially enrolled. Out of the 102 individuals who initially agreed to participate, 20 withdrew from the study prematurely and 14 died before being randomized to one of the three study groups. Ultimately, at baseline (T0), 68 patients and their partners were randomly assigned to either DT, DT accompanied by their partner (DT +), or SPC. At one-week follow-up (T1), 42 patients and their FCs participated, for a retention rate of 61.8%. The retention rate for FCs between T0 (baseline) and FU2 (3 months after their partner’s death) was 44%. The participation flow and loss to follow-up is depicted in Fig. 2. Conducing the DT intervention took 1.5 to 3 h for the Dignity therapists plus approximately 3 to 5 h for transcription and editing of the generativity document. The entire DT intervention, including passing the generativity document on to the patients, was ideally completed within 1–2 weeks.

### Acceptability

**Fig. 3:**
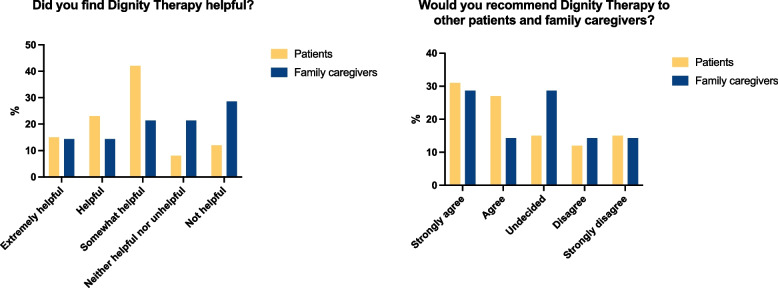
Patients’ and family caregivers’ experiences with dignity therapy

Dignity Therapy was well-received by the majority of patients and their FCs, as indicated by a mean score of 2.55 (SD = 1.19) on a 5-point Likert-scale (0 = not at all, 4 = very well received). Additionally, patients rated their likelihood of recommending the intervention to others with a mean score of 2.54 (SD = 1.45). Approximately two-thirds of patients (N = 28) indicated that DT helped them to review important life memories and that this reflection was transformed into thankful appreciation of life, general gratitude, and the discovery of greater sense, meaning, and purpose. Furthermore, patients perceived DT helpful for facilitating end-of-life conversations concerning their wishes and concerns and with decision-making with their FCs. Family caregivers also reported a positive impact of the intervention, rating perceived helpfulness with a mean score of 3.08 (SD = 1.30) (Fig. 3).

### Effects of dignity therapy intervention

There were no significant pre-post intervention changes for patients or their FCs, in terms of anxiety or depression levels, psychological distress, dignity-related distress, spiritual well-being, or suffering. When the two intervention groups (DT and DT +) were combined, comparing this coalesced group from pre- to post-intervention revealed a significant increase in patients’ perceived quality of life (FACIT-Pal-14) (mean difference 6.15, SD = 1.86, *p* < 0.01) (Table 2). No other outcome measure changed significantly over time in the DT/DT + intervention group. Group comparisons revealed a significant increase in the PRISM score (indicating less suffering) in the coalesced patient intervention group relative to controls (mean difference 1.55, SD 0.76, *p* < 0.05) (Table 3). A statistically significant interaction between group and time was observed for the patients’ total HADS score (F = 4.33, df = 1, 82.9; *p* = 0.04) and for the patients’ PRISM score (F = 7.99, df = 1,15.39; *p* = 0.01), indicating a protective effect of DT against further mental health deterioration, illness burden and suffering at follow-up (cf Tables 2, 3, and 4).

**Table 2:** Primary and secondary outcomes in patients: pre vs. post intervention (DT + and DT combined)

		**T0**			**T1**			**Comparison (T1-T0)**		**Statistics**
**Subgroups**	**Measures**	**N**	**Mean**	**SD**	**N**	**Mean**	**SD**	**Mean difference (SE)**	**95% CI**	***p-value***
DT + DT combined	Hastened Death	28	2.07	2.09	28	1.71	1.96	-0.36 (0.36)	-1.10; 0.38	0.33
	HADS _total_	28	17.04	6.09	28	16.96	7.35	-0.07 (0.62)	-1.34; 1.20	0.91
	HADS _anxiety_	28	8.50	3.36	28	8.29	4.23	-0.21 (0.40)	-1.03; 0.60	0.59
	HADS _depression_	28	8.54	3.64	28	8.68	4.33	0.14 (0.44)	-0.75; 1.04	0.75
	DTherm	28	5.79	2.54	28	6.21	2.32	0.43 (0.46)	-0.51; 1.36	0.36
	FACIT-Pal-14	27	27.67	5.88	27	33.81	7.84	6.15 (1.86)	2.33; 9.96	**0.01**
	FACIT-SP-12	27	24.42	4.44	27	25.54	9.50	0.12 (1.58)	-3.12; 3.37	0.94
	PDI tot	27	57.70	13.35	27	54.63	16.13	-3.07 (3.01)	-9.26; 3.12	0.32
	PDI _Symp distress_	27	2.44	0.66	27	240	0.84	-0.04 (0.14)	-0.33; 0.25	0.80
	PDI _Exi distress_	27	2.59	0.70	27	2.25	0.85	-0.33 (0.17)	-0.68; 0.02	0.06
	PDI _Dependency_	27	2.53	0.85	27	2.44	0.85	-0.10 (0.15)	-0.40; 0.21	0.52
	PDI _Peace of Mind_	27	1.88	0.73	27	1.77	0.70	-0.11 (0.18)	-0.48; 0.27	0.57
	PDI _Social Support_	27	1.47	0.75	27	1.44	0.73	-0.03 (0.13)	-0.30; 0.30	0.85
	PRISM	27	2.70	2.00	27	3.11	2.14	0.41 (0.23)	-0.05; 0.87	0.08
SPC	Hastened Death	13	2.00	1.78	13	1.92	1.98	-0.08 (0.40)	-0.95; 0.79	0.85
	HADS _total_	14	13.64	4.09	14	17.79	9.72	4.14 (2.61)	-1.501; 9.79	0.14
	HADS _anxiety_	14	6.14	2.60	14	8.36	4.81	2.21 (1.19)	-0.36; 4.79	0.09
	HADS _depression_	14	7.50	2.68	41	9.43	5.64	1.93 (1.53)	-1.37; 5.23	0.23
	DTherm	14	6.21	2.19	14	6.00	2.48	-0.21 (0.48)	-1.26; 0.83	0.66
	FACIT-Pal-14	11	30.82	5.33	11	30.90	9.47	0.08 (3.76)	-8.29; 8.46	0.98
	FACIT-SP-12	11	28.27	4.45	11	27.55	7.50	-0.73 (2.65)	-6.62; 5.17	0.79
	PDI tot	11	52.36	14.45	11	54.55	17.43	2.18 (2.81)	-4.07; 8.43	0.46
	PDI _Symp distress_	11	2.28	0.61	11	2.43	0.77	0.15 (0.21)	-0.31; 0.61	0.48
	PDI _Exi distress_	11	2.20	0.71	11	2.31	0.79	0.12 (0.11)	-0.13; 0.37	0.31
	PDI _Dependency_	11	2.25	0.87	11	2.22	0.83	-0.04 (0.13)	-0.32; 0.24	0.78
	PDI _Peace of Mind_	11	1.79	0.76	11	1.88	0.92	0.09 (0.23)	-0.43; 0.61	0.71
	PDI _Social Support_	11	1.42	0.54	11	1.48	0.72	0.06 (0.18)	-0.34; 0.46	0.74
	PRISM	13	4.25	2.68	13	2.79	1.91	-1.46 (0.84)	-3.30; 0.37	0.11

*Abbreviations:* *DT* + Dignity Therapy Patients and Partners, *DT* Dignity Therapy with Patients, *SPC* standard palliative care, *SD* standard deviation, *HADS* Hospital Anxiety and Depression Scale, *DTherm* Distress Thermometer, *FACIT-Pal-14* FACIT-Palliative Care, *FACIT-SP-12* FACIT Spiritual Well-Being, *PDI* Patient Dignity Inventory, *PRISM* Pictorial Representation of Illness and Self Measure

**Table 3:** Group comparison in patients (DT and DT + combined vs. SPC)

				**Comparison T1 – T0**		**Statistics**
**Measure**	**Time**	**Group**		**Mean difference (SE)**	**95% CI**	***p-value***
HADS_tot_	T0	DT and DT +	SPC	3.39 (1.81)	-0.26; 7.05	0.07
	T1	DT and DT +	SPC	-0.82 (2.68)	-6.24; 4.60	0.76
Hastened death	T0	DT and DT +	SPC	0.07 (0.67)	-1.290; 1.43	0.92
	T1	DT and DT +	SPC	-0.21 (0.66)	-1.54; 1.13	0.75
DTherm	T0	DT and DT +	SPC	-0.43 (0.79)	-2.04; 1.18	0.59
	T1	DT and DT +	SPC	0.21 (0.78)	-1.35; 1.78	0.78
FACIT-Pal-14	T0	DT and DT +	SPC	-3.15 (2.05)	-7.31; 1.01	0.13
	T1	DT and DT +	SPC	2.91 (2.98)	-3.13; 8.95	0.33
FACIT-SP-12	T0	DT and DT +	SPC	-2.85 (1.59)	-6.08; 0.37	0.08
	T1	DT and DT +	SPC	-2.00 (3.22)	-8.52; 4.52	0.54
PDI_tot_	T0	DT and DT +	SPC	5.34 (4.89)	-4.57; 15.25	0.28
	T1	DT and DT +	SPC	0.08 (5.90)	-11.89; 12.06	0.99
PRISM	T0	DT and DT +	SPC	-1.55 (0.76)	-3.08; -0.02	0.04
	T1	DT and DT +	SPC	0.32 (0.70)	-1.09; 1.74	0.65

*Abbreviations: DT* + Dignity Therapy Patients and Partners, *DT* Dignity Therapy with Patients, *SPC* standard palliative care, *SD* standard deviation, *HADS* Hospital Anxiety and Depression Scale, *DTherm* Distress Thermometer, *PRISM* Pictorial Representation of Illness and Self Measure

**Table 4:** Results of group-by-time interaction effects on all outcome measures

	**F**	**df**	***p***
HADS_tot_	4.33	1	**0.04**
Hastened death	0.22	1	0.64
DTherm	0.77	1	0.38
FACIT-Pal-14	2.60	1	0.12
FACIT-SP-12	0.08	1	0.78
PDI_tot_	1.08	1	0.31
PRISM	7.99	1	**0.01**

Family caregivers reported a significant increase in quality-of-life on the environmental subscale following the intervention (mean difference 4.44, SD 1.92, *p* < 0.05). They also had an increased PRISM score (indicating less suffering) post intervention (mean difference 1.67, SD 0.66, *p* < 0.05) (Table 1; cf supplementary material). No significant inter-group differences (intervention vs. control) or interaction effects (group x time) were found for FCs (Tables 2a and b; cf supplementary material).

## Discussion

The primary objectives of this study were to assess the feasibility and acceptability of DT interventions that included FCs of terminally-ill cancer patients receiving acute hospital care or hospice referrals. Our study was specifically designed to surmount methodological challenges and address gaps in the existing literature by using a randomized controlled trial, and exclusively enrolling patients with a significant baseline level of distress, as denoted by a total HADS score of eight or more.

Our findings indicate a strong level of patients’ and FCs’ acceptance for the DT intervention. Most study participants perceived the intervention as helpful, both for themselves and for their loved ones. Most of the individuals who completed the evaluation reported improvements in their emotional, spiritual well-being, illness burden, and suffering. These findings are consistent with previously-published results in cancer patients [14, 15, 30, 38]. This perspective is further solidified by our Dignity therapists, who reported personal satisfaction as a result of providing DT to their patients.

Unfortunately, the low participation rate and high attrition rate suggest that implementing the DT intervention as a randomized controlled trial (RCT) within the clinical context, is impracticable. These recruitment issues are likely due to the taxing demands associated with study participation, including pre- and post-intervention assessments with several questionnaires, lack of time, and our patients’ poor health status and/or rapid health decline approaching the end of life. It is possible that the low participation and high attrition rates may be linked more to the study design as an RCT and its inherent patient burden rather than to the DT intervention itself. Furthermore, over the course of our study, we encountered several challenges that have been previously reported in the literature [3, 14, 21, 25, 36]. One of the major obstacles was to identify the appropriate time window to administer the DT intervention. Despite the concerted efforts of our DT therapists, the fragile health status of the patients and their rapid disease progression at the end of life rendered the recruitment and enrollment of study participants nearly infeasible. Although DT was developed for patients nearing the end of their life [14], we conclude that such a DT intervention needs to be applied earlier in the disease trajectory. Additionally, in line with previous research [1], it was found that, for the Dignity therapists, conducting interviews, transcribing verbal content into written form, and the subsequent editing and delivery of the generativity document to the patients were highly time- and resource-intensive. Therefore, implementing DT in clinical settings with limited personnel available for administration and high patient volumes is virtually impossible.

### Effectiveness of dignity therapy

In our study, we found no statistically significant between-group differences in the levels of distress, anxiety, or depression among patients from before to after the intervention. Upon combining the two active intervention groups (DT and DT +), our analyses revealed that the DT intervention reduced suffering, conferred a sense of peace, and demonstrated a protective effect against further mental health deterioration at follow-up in patients. However, given the limited sample size, the high attrition rate, multiplicity issues in clinical trials as well as the short follow-up period of only one week, caution is necessary when interpreting this outcome. Moreover, it is possible that disease progression, as well as the effects of cancer therapy might reverse improvements in well-being following DT and that improvements might subside relatively quickly over time.

Family caregivers reported improved quality of life on the environmental subscale and a reduction in suffering three months after their ill partner’s death. The death of a loved one can be an incredibly difficult experience, causing immense pain, grief, and suffering that may last for a long time. However, over time, some individuals may experience personal growth, improved quality of life, and reduced suffering [48]. Losing a loved one can sometimes help people gain clarity about their own lives. Experiencing a loved one’s death also can help one to appreciate the things they have, cherish their relationships, pursue changes or different goals, and focus on what truly matters to them. However, it remains difficult to determine whether such changes would be the result of a DT intervention or the effects of time and healing from loss and grief.

Clearly, the inclusion of FCs in the DT intervention allowed patients to speak about things that matter to them, express appreciation, and share valuable experiences in the presence of their loved ones. This exchange of memories, thoughts, and emotions may help FCs to cope with their loved one’s end-of-life situation and potentially facilitate bereavement. Thus, investigating the effectiveness of DT interventions that incorporate patients' partners or family members is a crucial area for future research.

### The importance of family involvement in dignity therapy

Interestingly, despite the acknowledged importance of family and communication in maintaining terminally-ill patients' sense of dignity, dignity-promoting interventions often neglect the involvement of family caregivers [58]. Effective communication is crucial to promoting mutual understanding, providing emotional support, and strengthening human connections, all of which are vital for supporting patients nearing the end of their life, and their loved ones [28]. Dignity Therapy is an evidence-based intervention that facilitates meaningful conversations among patients and their loved ones, promoting emotional disclosure and enhancing family connections and relationships [11]. Including a loved one in DT sessions can help patients to feel more connected to their life and legacy, enhancing their sense of self-worth and perceived meaning [57]. Our descriptive data suggest that partners and caregivers also benefit from a DT intervention, corroborating findings already reported in the literature [46]. A recent study further demonstrated that such interventions can alleviate family caregivers’ anxiety and depression [55].

Family involvement in DT is a critical aspect of promoting a more personalized and meaningful experience for terminally-ill patients, and for supporting both patients and their loved ones during the end-of-life process. Moreover, understanding a patient's values and wishes empowers FCs to provide more meaningful support during the patient's final days. Importantly, family involvement in DT may also facilitate the bereavement process by helping loved one’s cope with loss and grief.

### Clinical implications

Communication at the end of life is imperative. It not only relieves stress, but improves the dying individual’s sense of well-being, facilitates meaningful interactions, and promotes patient-centered care, resulting in more satisfying outcomes for all involved [41]. However, discussing one’s needs or concerns regarding end-of-life care or hopes and wishes for the family can be difficult due to societal taboos around death and dying, making it a challenging topic to confront [9]. In addition, many FCs feel overwhelmed by the end-of-life situation; and the rapid disease progression of their loved one often leaves them at a loss for words or questions to ask.

Balancing the benefits of a DT intervention with its constraints, we suggest offering a slightly modified and less time- and resource-intensive version of DT. One such option is to provide the DT inventory – a semi-structured interview guide – to partners, family members, and other caregivers of terminally-ill patients, enabling them to ask the questions that matter to them most and document the patients' memoirs by employing whatever means are available to them. Using a Dignity inventory may facilitate meaningful communication between those who are dying and their loved ones. Final conversations may deepen connections, providing a sense of closure in relationships and helping FCs to begin the grieving process while the dying loved one is still present. Such interactions can facilitate the process of bereavement and coping with grief and loss, enabling grieving FCs to grow from this experience and move towards acceptance and healing [29].

### Strengths and limitations

Our results should be interpreted in light of several study limitations. Firstly, the study was restricted by its small sample size. As a consequence, the anticipated number of patients calculated in the power analysis was not reached, potentially hindering the ability to detect subgroup effects. This shortfall increases the risk of false subgroup effects or amplifies the potential for spurious significant results due to chance [7]. Second, the low participation and the high attrition rate poses an additional constraint on our study, thereby further limiting reliability and interpretation of our results. High attrition rates are common in studies involving individuals who suffer from advanced terminal illnesses, as these individuals typically experience high symptom burdens and the side effects of therapy [23]. In addition, the study’s RCT design, alongside its inherent weaknesses, including patient burden associated with data collection procedures (*i.e.,* pre- and post-intervention assessments with several questionnaires) might have been perceived as onerous by certain study participants. This perception could potentially account for their withdrawal from the study, contributing in part to the observed high attrition rate. Third, it is important to note that floor effects for the primary outcome (HADS ≥ 8) could potentially exert a substantial influence on the outcome findings and the interpretation of the study results. Such effects may also impact the validity and reliability of our findings. A further limitation inherent to our study pertains to the consideration of multiple outcomes and analyses (multiplicity). It is important to acknowledge that conducting multiple tests increases the likelihood of chance findings. To mitigate this concern and in reference to [31], we employed a group*time interaction term into our analyses. Additionally, due to the short follow-up period of only one week between our baseline and patient follow-up, this study was unable to demonstrate any sustained effect of dignity therapy on psychological distress, anxiety, depression, or spiritual well-being over time. Although future research could be strengthened by longer-term follow-up with patients, it is doubtful that a long-term protocol would be feasible in this particularly vulnerable group of terminally-ill patients. Furthermore, maintaining a positive health effect following DT will be difficult in a population of terminally-ill patients who often suffer from anxiety and depression as a result of disease progression and increasing symptom burden and functional impairment [50].

Despite its limitations, our study also has several important strengths. Firstly, the rigorous methodology employed in this study, which included a randomized controlled design conducted over a 7-year period, and ensuring that only individuals with a minimum total HADS score of eight were considered eligible for the study. This approach enhanced the internal validity of our findings, minimizing the possibility of confounding variables affecting the results. Additionally, the data collection was conducted across multiple study centers, increasing the external validity of the study, and improving the generalizability of our results. Consequently, our findings are likely to be applicable to a broader population beyond the specific study sample.

## Conclusions

Dignity therapy has the potential to benefit terminally-ill patients and their loved ones. Our study’s findings strongly support the acceptability of DT, with or without direct partner/caregiver involvement, among cancer patients and their FCs. The DT intervention is a useful tool to enable end-of-life conversations among terminally-ill patients and their loved ones. It provides opportunities for patients to reminisce and consider what matters most to them, and to convey gratitude, wishes, and guidance to their loved ones. However, implementing the DT intervention is time-intensive and its feasibility in clinical settings may be limited by the current chronic lack of resources. Therefore, we suggest a slightly modified and less resource-intensive version of DT. One such option would be to provide the DT inventory to FCs of terminally-ill patients, facilitating end-of-life conversations, and empowering everyone to ask the questions that matter most to them.

### Supplementary Information

**Supplementary Material 1.**

**Supplementary Material 2.**

## Data Availability

The datasets generated and/or analyzed during the current study are not publicly available due to ethics restrictions but are available from the corresponding author on reasonable request.

## Abbreviations

DT: Dignity Therapy
DT +: Dignity Therapy including their FCs
DTherm: The Distress Thermometer
FACIT-SP: Functional Assessment of Chronic Illness Therapy Palliative Care (FACIT-Pal) and Spiritual Wellbeing Scale
FCs: Family caregivers
HADS: The Hospital Anxiety and Depression Scale
HRQoL: Health-related quality of life
ITT: Intention-to-treat analysis
KEK: Ethics Committee of the Canton Zurich, Switzerland
M: Mean
MAID: Medical aid in dying
PDI-G: Patient Dignity Inventory—German Version
PRISM: Pictorial Representation of Illness and Self Measure
RCT: Randomized controlled trial
SD: Standard deviation
SPC: Standard palliative care
SPSS: IBM Statistical Package for the Social Sciences
WHOQOL-BREF: WHO Quality of Life Questionnaire
