# Supplementary material for: Effects of dignity therapy on psychological distress and wellbeing of palliative care patients and family caregivers – a randomized controlled study
Source: BMC Palliat Care. 2024 Mar 14;23:73. doi: 10.1186/s12904-024-01408-4 (PMC10938771; doi:10.1186/s12904-024-01408-4)
Supplement: Supplementary file 2 — Supplementary Material 2. [file 12904_2024_1408_MOESM2_ESM.docx]

**Supplementary Tables**

**Table 1.** Primary and secondary outcomes in family caregivers: pre vs. post intervention: FU2 – T0 (DT+ and DT combined)

|  |  | **T0** | | |  | **FU2** | | |  | **Comparison**  **(T3-T0)** | | **Statistics** |
| --- | --- | --- | --- | --- | --- | --- | --- | --- | --- | --- | --- | --- |
| **Subgroups** | **Measures** | **N** | **Mean** | **SD** |  | **N** | **Mean** | **SD** |  | **Mean difference (SE)** | **95% CI** | ***p-value*** |
| DT + DT combined | HADS _total_ | 19 | 14.47 | 7.77 |  | 19 | 12.79 | 8.25 |  | -1.68 (1.42) | -4.664; 1.296 | 0.251 |
|  | HADS _anxiety_ | 19 | 8.42 | 4.28 |  | 19 | 6.58 | 4.13 |  | -1.84 (0.89) | -3.704; 0.020 | 0.052 |
|  | HADS _depression_ | 19 | 6.05 | 4.08 |  | 19 | 6.21 | 4.61 |  | 0.16 (0.77) | -1.783; 1.467 | 0.841 |
|  | Dtherm | 19 | 5.79 | 2.76 |  | 19 | 4.95 | 2.80 |  | -0.84 (0.75) | -2.410; 0.726 | 0.274 |
|  | WHOQOL _global_ | 19 | 63.16 | 23.00 |  | 19 | 69.08 | 20.57 |  | 5.92 (3.50) | -1.422; 13.264 | 0.107 |
|  | WHOQOL _phys_ | 19 | 69.71 | 21.31 |  | 19 | 69.36 | 18.07 |  | -0.35 (3.20) | -7.063; 6.374 | 0.915 |
|  | WHOQOL _psych_ | 19 | 65.35 | 20.46 |  | 19 | 62.89 | 18.24 |  | -2.46 (4.46) | -11.742; 6.830 | 0.585 |
|  | WHOQOL _social_ | 19 | 61.40 | 23.93 |  | 19 | 65.57 | 18.42 |  | 4.17 (3.85) | -3.377; 11.711 | 0.261 |
|  | WHOQOL _env_ | 19 | 75.49 | 14.22 |  | 19 | 79.93 | 17.47 |  | 4.44 (1.92) | 0.414; 8.467 | **0.032** |
|  | PRISM | 18 | 2.48 | 1.41 |  | 19 | 4.16 | 2.81 |  | 1.67 (0.66) | -0.285; 3.060 | **0.021** |
|  |  |  |  |  |  |  |  |  |  |  |  |  |
| SPC | HADS _total_ | 16 | 14.88 | 8.00 |  | 16 | 15.44 | 8.42 |  | 0.56 (0.94) | -1.431; 2.556 | 0.557 |
|  | HADS _anxiety_ | 16 | 8.38 | 4.46 |  | 16 | 8.56 | 4.52 |  | 1.89 (0.59) | -1.077; 1.452 | 0.756 |
|  | HADS _depression_ | 16 | 6.50 | 4.03 |  | 16 | 6.88 | 4.40 |  | 0.38 (0.47) | -0.634; 1.384 | 0.440 |
|  | Dtherm | 16 | 5.81 | 2.79 |  | 16 | 5.75 | 2.49 |  | -0.06 (0.50) | -1.119; 0.994 | 0.901 |
|  | WHOQOL _global_ | 15 | 70.83 | 18.09 |  | 15 | 61.68 | 22.39 |  | -9.17 (5.24) | -20.410; 2.077 | 0.102 |
|  | WHOQOL _phys_ | 15 | 74.05 | 13.39 |  | 15 | 73.57 | 15.79 |  | -0.48 (1.70) | -4.128; 3.176 | 0.784 |
|  | WHOQOL _psych_ | 15 | 63.89 | 11.64 |  | 15 | 62.83 | 14.96 |  | -1.06 (1.81) | -4.930; 2.819 | 0.568 |
|  | WHOQOL _social_ | 15 | 61.09 | 21.36 |  | 15 | 63.10 | 13.76 |  | 1.19 (4.61) | -8.770; 11.151 | 0.800 |
|  | WHOQOL _env_ | 15 | 78.35 | 11.59 |  | 15 | 81.70 | 9.48 |  | 3.35 (1.98) | -0.925; 7.622 | 0.114 |
|  | PRISM | 17 | 3.25 | 2.05 |  | 15 | 3.03 | 1.89 |  | -0.21 (0.24) | -0.723; 0.296 | 0.384 |

*Abbreviations.* FU2, Follow-up 2; DT+, Dignity Therapy Patients and Partners; DT, Dignity Therapy with Patients; SPC, standard palliative care; SD, standard deviation; HADS, Hospital Anxiety and Depression Scale; Dtherm, Distress Thermometer; PRISM, Pictorial Representation of Illness and Self Measure.

**Table 2a.** Group comparison family caregivers FU2 – T0 (DT and DT+ combined vs. SPC)

|  |  |  |  | **Comparison FU2 – T0** | | **Statistics** |
| --- | --- | --- | --- | --- | --- | --- |
| **Measure** | **Time** | **Group** | | **Mean difference (SE)** | **95% CI** | ***p-value*** |
| HADS_tot_ | T0 | DT and DT+ | SPC | -0.89 (3.02) | -7.076; 5.297 | 0.770 |
|  | FU2 | DT and DT+ | SPC | 1.88 (2.77) | -3.789; 7.549 | 0.502 |
| Dtherm | T0 | DT and DT+ | SPC | -0.30 (1.03) | -2.417; 1.814 | 0.773 |
|  | FU2 | DT and DT+ | SPC | -0.05 (1.00) | -2.090; 1.985 | 0.958 |
| WHOQOL _global_ | T0 | DT and DT+ | SPC | -7.30 (8.37) | -24.445; 9.852 | 0.391 |
|  | FU2 | DT and DT+ | SPC | -5.92 (7.20) | -20.672; 8.830 | 0.418 |
| PRIMS | T0 | DT and DT+ | SPC | -0.09 (0.54) | -1-204; 1.030 | 0.875 |
|  | FU2 | DT and DT+ | SPC | -1.39 (0.98) | -3.408; 0.637 | 0.171 |

*Abbreviations*. FU2, Follow-up 2; DT+, Dignity Therapy Patients and Partners; DT, Dignity Therapy with Patients; SPC, standard palliative care; SD, standard deviation; HADS, Hospital Anxiety and Depression Scale; Dtherm, Distress Thermometer; Assessment Scale; PRISM, Pictorial Representation of Illness and Self Measure.

**Table 2b.** Results of group-by-time interaction effects on all outcome measures

|  | **F** | **df** | ***p*** |
| --- | --- | --- | --- |
| HADS_tot_ | 1.245 | 1 | 0.274 |
| DTherm | 0.046 | 1 | 0.832 |
| WHOQOL _global_ | 0.060 | 1 | 0.808 |
| PRIMS | 2.414 | 1 | 0.132 |
